# IgG-Containing Isoforms of Neuregulin-1 Are Dispensable for Cardiac Trabeculation in Zebrafish

**DOI:** 10.1371/journal.pone.0166734

**Published:** 2016-11-15

**Authors:** Leigh Ann Samsa, Cade Ellis Ito, Daniel Ross Brown, Li Qian, Jiandong Liu

**Affiliations:** 1 Department of Cell Biology and Physiology, University of North Carolina at Chapel Hill, Chapel Hill, North Carolina, United States of America; 2 McAllister Heart Institute, University of North Carolina at Chapel Hill, Chapel Hill, North Carolina, United States of America; 3 Department of Pathology and Laboratory Medicine, University of North Carolina at Chapel Hill, Chapel Hill, North Carolina, United States of America; Academia Sinica, TAIWAN

## Abstract

The Neuregulin-1 (Nrg1) signaling pathway has been widely implicated in many aspects of heart development including cardiac trabeculation. Cardiac trabeculation is an important morphogenetic process where clusters of ventricular cardiomyocytes extrude and expand into the lumen of the ventricular chambers. In mouse, Nrg1 isoforms containing an immunoglobulin-like (IgG) domain are essential for cardiac trabeculation through interaction with heterodimers of the epidermal growth factor-like (EGF-like) receptors ErbB2/ErbB4. Recent reports have underscored the importance of Nrg1 signaling in cardiac homeostasis and disease, however, placental development has precluded refined evaluation of the role of this pathway in mammals. ErbB2 has been shown to have a developmentally conserved role in cardiac trabeculation in zebrafish, a vertebrate model organism with completely external development, but the requirement for Nrg1 has not been examined. We found that among the multiple Nrg1 isoforms, the IgG domain-containing, type I Nrg1 (*nrg1-I*) is the only isoform detectable in the heart. Then, using CRISPR/Cas9 gene editing, we targeted the IgG domain of Nrg1 to produce novel alleles, *nrg1*^*nc28*^ and *nrg1*^*nc29*^, encoding *nrg1-I* and *nrg1-II* truncations. Our results indicated that zebrafish deficient for *nrg1-I* developed trabeculae in an ErbB2-dependent manner. Further, these mutants survive to reproductive adulthood with no overt cardiovascular defects. We also found that additional EGF-like ligands were expressed in the zebrafish heart during development of trabeculae. Together, these results suggest that Nrg1 is not the primary effector of trabeculation and/or that other EGF-like ligand(s) activates the ErbB2/ErbB4 pathway, either through functioning as the primary ligand or acting in a redundant manner. Overall, our work provides an example of cross-species differences in EGF family member requirements for an evolutionary conserved process.

## Introduction

Congenital heart diseases (CHD) are highly prevalent birth defects [1] and often feature perturbations in cardiac morphogenesis that arise during development [2–4]. The Nrg1-ErbB2/4 signaling pathway has been implicated in many aspects of vertebrate cardiac biology ranging from heart development to homeostasis and disease [5]. Transmembrane pro-Nrg1 is expressed on endocardial and microvascular endothelial cells where it is cleaved by extracellular secretases to release active Nrg1 [6–9]. Once cleaved, Nrg1 binds to cardiomyocyte-expressed ErbB4 via its epidermal growth factor (EGF) domain. This ligand and receptor interaction subsequently stimulates hetero-dimerization of ErbB4 with its essential co-receptor ErbB2, leading to activation of ErbB2 tyrosine kinase activity and downstream signaling [10–12]. Recent reports have underscored the importance of the Nrg1-ErbB2/4 signaling pathway in cardiac repair processes, and recombinant Nrg1 is currently in clinical trials as a heart failure therapeutic, but the role of Nrg1 in development is largely unknown [5,13–19]. A refined understanding of this role could provide insight into CHDs or inform the development of improved therapeutics.

Early studies demonstrated that Nrg1, ErbB2 and ErbB4 are each required for proper chamber maturation and cardiac development in mice [7,20–22]. Owing to their rapid development, optical clarity, and ease of genetic manipulation, zebrafish (*Danio rerio*) have emerged as a premier model organism for understanding the molecular and genetic regulation of heart development [23]. Unlike mammalian models, zebrafish embryos are small enough to meet oxygen needs by diffusion alone and can survive for days with severe heart malformations [24–28]. Further, adult zebrafish are highly tolerant of reduced cardiac function [29,30].

The early embryonic zebrafish heart develops into a two-chambered heart within 48 hours post-fertilization (hpf). As the heart matures, it optimizes the ventricular myocardial architecture for efficient conduction and contraction. This chamber maturation features formation of highly organized luminal myocardial protrusions called trabeculae, which are evident by 72 hpf and comprise the majority of the adult myocardium [31–33]. Failure to initiate trabeculation is embryonic lethal in mice and zebrafish, and trabeculation defects are often associated with CHDs [34,35]. Though our previous work demonstrated that ErbB2 is required for zebrafish cardiac trabeculation, requirement for Nrg1 in zebrafish cardiac development has not been examined [31].

Nrg1 is alternatively spliced to produce a diversity of isoforms [36]. Zebrafish *nrg1* produces three major isoforms by alternative splicing, *nrg1-I*, *nrg1-IIa-c*, and *nrg1-III*. The N-terminus contains either a cysteine-rich domain (*nrg1-III*) or a unique N-terminal sequence followed by an IgG-like domain (*nrg1-I* and *nrg1-II*). All isoforms share an EGF-like domain, a transmembrane domain, and a C-terminal Neuregulin domain. In mice, genetic deletion of the IgG domain-containing isoforms (*Nrg1-I* and *Nrg1-II*) is sufficient to block cardiac trabeculation [7,21].

To determine the genetic requirement for Nrg1 in zebrafish trabeculation, we used CRISPR/Cas9 targeted nuclease activity to generate frameshift mutations that lead to early truncation of IgG-containing Nrg1-I and Nrg1-II. The mutant embryos had reduced *nrg1* transcripts levels, suggesting non-sense mediated decay and absence of previously unannotated splice isoforms. Yet, the mutant fish survived to adulthood without cardiac trabecular defects or overt signs of other cardiac malformations. This lack of cardiac phenotypes in zebrafish *nrg1-I*/*II* mutants may be explained by expression of other putative EGF-like ligands expressed in the developing heart. Together, these results suggest that Nrg1 is dispensable for heart development in zebrafish and that additional mutants will need to be generated to determine which ligand(s) have the primary role of regulating trabeculation or if other ligands play compensatory or redundant roles in trabeculation.

## Materials and Methods

### Animal Lines and Care

Embryos and adult fish were reared and maintained at the aquaculture facility of the University of North Carolina at Chapel Hill at 28.5°C on a 14h/10h light/dark cycle in accordance with the University of North Carolina at Chapel Hill Institutional Animal Care and Use Committee (IACUC) approved protocol [37]. All studies were performed after euthanasia of zebrafish and all efforts were made to minimize animal suffering. The zebrafish lines used in this study are as follows: *nrg1*^*z26*^ [38], *nrg1*^*nc28*^, *nrg1*^*nc29*^, *Tg*(*myl7*:*dsRed*)^*vc6*^ [39], *Tg(kdrl*:*EGFP)*^*s843*^ [40], and *Tg(myl7*:*GFP)*^*twu26*^ [41].

### CRISPR/Cas9 design and injection

Cas9 mRNA was *in vitro* transcribed from pXT7 using the mMessage mMachine kit (ThermoFisher) as previously described with some modifications [42]. CRISPR/Cas9 target site in exon 3 of *nrg1* were identified using ZiFit software [43] and zebrafish genomic sequence, build GRCz9 [44]. Single stranded oligonucleotides corresponding to the targeting sequence were annealed and cloned into the DR274 vector (Addgene) [45], then transcribed *in vitro* with T7 MaxiScript kit (ThermoFisher). Cas9 plasmid was generously provided by Dr. Jing-Wei Xiong. Embryos were injected at the one cell stage with 1–2 nl of a mixture containing 1200 ng Cas9, 50–75 ng gRNA, 10 mM MgCl, and 0.01% phenol red. gRNA targeting efficiency was determined by High Resolution Melt Analysis (HRMA) [46] as described below using primers flanking the target site. F1 offspring from F0 founders that carry favorable mutations, determined by DNA sequencing of the target site, were raised to adulthood. Heterozygous F1 fish were interbred to produce homozygous wild type, homozygous mutant, and heterozygous mutant offspring.

### Genotyping

#### Genomic DNA isolation

Genomic DNA was collected from fin clips or embryos in 50 or 25 μL lysis buffer containing 10 mM Tris-HCl pH 8.0, 50 mM KCl, 0.3% Tween-20. Samples were lysed at 95°C for 10 minutes, and then digested in 0.5 μg/mL Proteinase K (Denville Scientific).

#### Line-specific genotyping

*nrg1*^*z26*^ fish were genotyped by PCR and enzyme digestion as previously described [38]. HRMA was used to genotype wild type, mutant, and heterozygous *nrg1*^*nc28*^ and *nrg1*^*nc29*^ lines (see below). Heterozygous alleles had multiple peaks in the derivative melt curve. Homozygous wild type and mutant allele melt temperatures differed by at least >1°C. This genotyping method was verified both by enzyme digestion and by DNA sequence analysis in a subset of samples.

### HRMA

High resolution melt analysis (HRMA) was used to validate CRISPR/Cas9 reagents, identify F1 founders, and genotype *nrg1*^*nc28*^ and *nrg1*^*nc29*^ fish. Each 10 μl reaction contained 0.5 μl genomic DNA (see above), 5 μl SYBR Green (ThermoFisher), and 4.5 μl primer mix (water with 0.7 mM forward and reverse primers). Fluorescence was measured every 0.025°C in a melt curve from 55–95°C. HRMA peaks were called from the derivative curve using a ViiA7 qRT-PCR machine equipped with HRMA package (ThermoFisher).

### PCR and qRT-PCR

RNA was isolated from whole embryos using Trizol reagent (Thermofisher) and from embryonic hearts using Qiagen RNAeasy Mini Plus Kit according to manufacturer’s instructions. Up to 1 μg of cDNA was reverse transcribed using Superscript Master Mix (Thermofisher). GoTaq (Promega) reagents were used for PCR with 10 ng cDNA template as per manufacturer’s instructions. For qRT-PCR, we used Syber Green chemistry (ThermoFisher) on a ViiA7 qPCR machine in 10 μL reactions. Cycle threshold (CT) values were normalized to *ef1a* as a housekeeping gene and relative expression was calculated comparing average change in CT in wild type and mutant embryos by the 2^^(ΔΔCT)^ method [47].

### Heart isolations

Heart isolations were performed as previously described [48]. Briefly, larvae were euthanized with 5X Tricaine at 3 dpf (days post-fertilization). Fine forceps were used to manually remove each heart (ventricle, atrium, and bulbous arteriosus) and dissect away non-cardiac tissues. Hearts were transferred to lysis buffer and processed according to manufacturer’s instructions for the RNAeasy Mini Plus kit (Qiagen). A minimum of 30 hearts were pooled for each gene expression replicate.

### FACS

Fluorescence activated cell sorting (FACS) of endothelial and myocardial cells was performed essentially as previously described with minor modifications [49]. Briefly, *Tg(kdrl*:*EGFP)*^*s843*^ or *Tg(myl7*:*GFP)*^*twu26*^ embryos were dissociated into single cells by enzymatic digestion, then counterstained with SYTOX Blue dead cell stain (ThermoFisher). *Tg(myl7*:*GFP)*^*twu26*^ embryos were passed through a 21 gauge needle 100 μm cell strainer to enrich for hearts prior to an abbreviated enzymatic digestion step. For each of 3–6 replicates, 2000–10,000 live, GFP+ cells were sorted with Sony SH800S then processed for qRT-PCR as described above using the Qiagen RNA Easy Micro kit (Qiagen).

### In situ hybridization

*In situ* hybridization was performed as previously described [50]. *In situ* hybridization probe for *nrg1* was prepared as previously described [8] and synthesized from the pGEMT vector (Promega) using the DIG RNA labeling kit (Roche). Whole-mount embryo imaging was performed on a Leica MZ16F fluorescence stereomicroscope.

### Confocal microscopy

Anesthetized larvae 2–5 dpf were embedded in 1% low melt agarose and oriented for optimal viewing of the heart. Immediately prior to imaging, embryos were euthanized with 5-10X Tricaine (MS-222, Sigma). After cessation of heartbeat, confocal z-stacks were collected using an Olympus Fluoview 1000MPE equipped with a 20X XLPlan water immersion objective (NA 1.0) with 2.5X optical zoom. Fluoview software was used to collect sections through the middle 25–50% of the heart at 512x512 or 1024x1024 pixel resolution and 1–2 μm spacing between z-slices. Fluoview’s brightness correction algorithm was used to account for signal attenuation with increasing depth. ImageJ [51] was used to process images. For each Z-stack, we selected either a maximum projection image of the whole stack or a representative mid-chambers slice for the appropriate analysis. Confocal data was collected for a minimum of 3 embryos for each condition, with matching controls for each experiment, where the N>3 embryos were selected as the representative samples from a pool of a minimum of N>12 embryos which were visually inspected for phenotype.

### Whole mount microscopy

Adult fish were anesthetized with Tricaine in system water. Fish were imaged alongside a centimeter ruler in a minimal volume of water using an Android 13 MP camera. Brightness and contrast were adjusted and images were scaled using ImageJ software.

### Mitotracker assay

Supernumerary neuromasts were assayed essentially as previously described [52]. Briefly, larvae were incubated for 5–30 minutes in fish water containing Mitotracker Red (ThermoFisher) at a 1:10,000 dilution. Larvae were briefly rinsed with system water and anesthetized with 1X Tricaine, then oriented in a lateral position and epifluorescence images were collected on a Leica M205C fluorescence stereoscope. The number of neuromasts on the lateral line were counted for N>25 embryos. Wild type embryos had 8–12 neuromasts, and we considered 18+ neuromasts to be supernumerary.

### Histology

Adult fish were euthanized on ice for 20 minutes. To ensure rapid and complete fixation, each fish was gavaged with 4% paraformaldehyde (PFA) in PBS, then the abdominal cavity was opened by anterior-posterior incision and flushed with 4% PFA. After overnight fixation, the fish were de-calcified with 0.5M EDTA for 3–7 days, dehydrated in 70% ethanol, paraffin embedded and sectioned at 5 μm intervals and stained with hematoxolin and eosin (H&E).

### Survival curve

Embryos were obtained from breeding healthy homozygous *nrg1*^*WT/WT*^ and *nrg1*^*nc28/nc28*^ adults. For each genotype, 7 tanks containing 10 fish each were raised under standard husbandry conditions. Tank order was randomized to minimize husbandry position effects. Survival was recorded weekly at 6–8 day intervals through 12 wpf (weeks post-fertilization).

### PD168393 treatment

Embryos were treated with 3.75 μM PD168393 (ThermoFisher) in 1% DMSO containing embryo medium from 2 dpf to 4 dpf. Control embryos were incubated in 1% DMSO in embryo medium.

## Results

### Molecular features of zebrafish Nrg1 and its expression in the zebrafish heart

**Fig 1:**
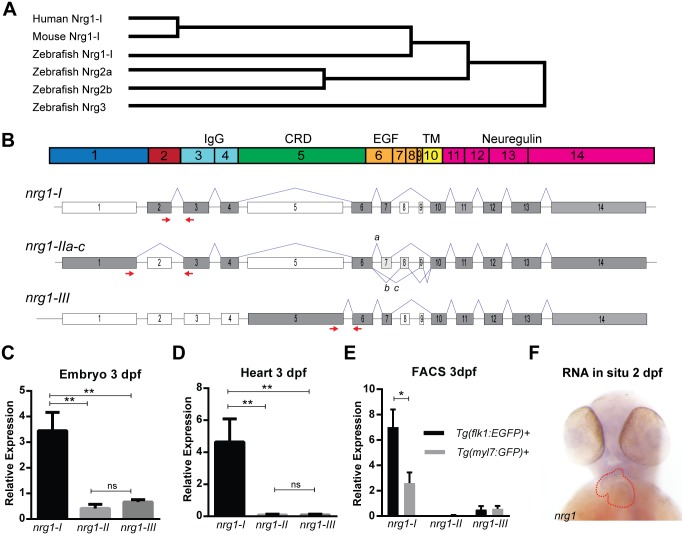
Zebrafish Neuregulin 1 and the expression of its isoforms. (A) Gene tree from Clustal-Omega multiple alignment comparison. (B) Schematic of Nrg1 domains encoded by Exons 1–14. (C) Schematic of nrg1 gene structure. Exons are drawn to scale; introns are not to scale. Alternative splicing produces three primary isoforms, *nrg1-I*, *nrg1-IIa-c*, and *nrg1-III*. (D-E) Relative expression of *nrg1* isoforms in (D) 3 dpf embryos and (E) dissected hearts from 3 dpf embryos normalized to *efl1a*. (F) In situ hybridization of anti-sense riboprobe targeting *nrg1*. Heart is outlined in red. Student’s T-test compared to matched control. Error bars are SEM. N≥3 biological replicates. *p≤0.05–0.01, **p≤0.01–0.001, ***p<0.001.

The zebrafish genome encodes several members of the neuregulin family—*nrg1*, *nrg2a*, *nrg2b*, and *nrg3*. *nrg1*, which encodes the putative ligand for cardiac ErbB2 signaling, may be an important regulator of trabeculation in zebrafish. Sequence analysis further indicates that zebrafish Nrg1 is the closest homolog to human NRG1 and mouse Nrg1 (Fig 1A). In the zebrafish genome, *nrg1* is located on Chromosome 18 and is predicted to have 14 coding exons encoding several functional domains (Fig 1B). Alternative splicing of *nrg1* produces 3 primary isoforms (*nrg1-I*, *nrg1-II*, and *nrg1-III*, Fig 1C) that differ primarily in their N-terminal sequence and lead to differential representation of functional domains. An immunoglobulin (IgG) domain is found in the N-terminus of *nrg1-I* and *nrg1-II* while a membrane-spanning cysteine-rich domain (CRD) is found in the N-terminus of *nrg1-III* (Fig 1B and 1C). In addition, all isoforms share an epidermal growth factor-like domain (EGF), a transmembrane domain (TM), and a C-terminal neuregulin domain (Fig 1B and 1C).

To determine which isoform(s) of *nrg1* are expressed in the heart, we designed exon-spanning primers to assess relative expression levels of *nrg1-I*, *nrg1-II*, and *nrg1-III* at 3 dpf. All three isoforms were detectable in whole embryo (Fig 1D). However, only *nrg1-I* was detectable in cardiac tissue (Fig 1E). We detected *nrg1* by *in situ* hybridization in the heart and brain of embryo (Fig 1F). Previous studies suggest that cardiac *nrg1* expression is confined to endocardial cells in the embryo [35,53,54]. Consistently, using FACS-enriched cells from a preparation of 3 dpf hearts, we also found that in zebrafish, *nrg1-I* was expressed in the endocardial but not myocardial cells (Fig 1E).

### Generation of zebrafish mutant alleles

**Fig 2:**
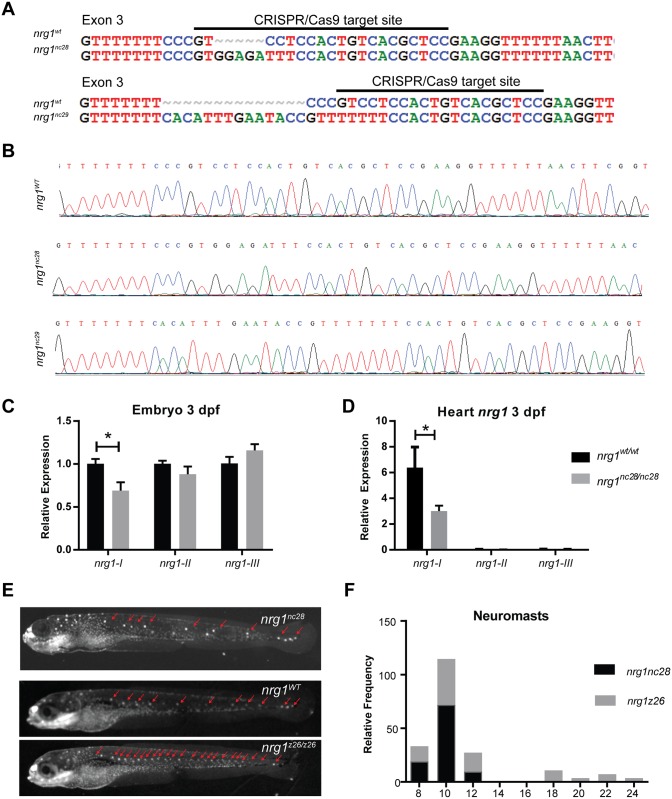
Zebrafish *nrg1-I/II* mutants. (A) CRISPR/Cas9 gene targeting and validation of *nrg1*^*nc28*^ and *nrg1*^*nc29*^ alleles showing target site and mutations. (B) Sanger sequence of *nrg1*^*WT*^, *nrg1*^*nc28*^ and *nrg1*^*nc29*^ alleles spanning target site in Exon 3. (C) Gel electrophoresis and densitometry of *nrg1* amplified from 10ng cDNA derived from *nrg1*^*WT*^ or *nrg1*^*nc28/nc28*^ embryos at 5 dpf. Student’s T-test compared to matched control. Error bars are SEM. N≥3 biological replicates. *p≤0.05–0.01. (D) Representative Mitotracker stain for neuromasts. Heterozygous adult fish carrying *nrg1*^*WT/nc28*^, *nrg1*^*WT/nc26*^, or *nrg1*^*WT/z26*^ alleles were inbred, and resulting offspring were evaluated for supernumerary neuromasts (red arrows). (E-F) Frequency distribution of the number of neuromasts per embryo. Blue bar marks range of neuromasts found in wild type larvae; red bars mark supernumerary neuromasts. Similar results were obtained with *nrg1*^*nc29*^ lines (data not shown). N = 15–20 embryos imaged per pairing; N = 2 biological replicates.

To investigate the isoform-specific role of Nrg1 in heart development, we used CRISPR/Cas9 gene editing to target its exon 3, which encodes part of the IgG domain shared by *nrg1-I* and *nrg1-II*. We isolated two frameshift alleles, *nrg1*^*nc28*^ and *nrg1*^*nc29*^, carrying a 5 bp insertion and 14 bp deletion in exon 3, respectively (Fig 2A and 2B). These mutations are predicted to truncate Nrg1-I at 55 and 99 amino acids upstream of the receptor binding EGF-like domain (S1 Fig). Interbreeding heterozygous fish for both alleles produced homozygous and heterozygous alleles at expected Mendelian ratios.

Since *nrg1*^*nc28*^ truncation was predicted to be more severe than the *nrg1*^*nc29*^ allele, we focused our efforts on phenotyping this allele. *nrg1* mRNA expression levels were dramatically reduced relative to wild type embryos, suggesting nonsense mediated decay of the mutant transcript (Fig 2C). To further determine the effect of frameshift mutation on *nrg1* expression level, *nrg1* mRNA expression level was measured via qPCR since loss of function mutant transcripts can have decreased stability through nonsense mediated decay. Transcript level of *nrg1* in the mutant was significantly reduced relative to that in the wild type embryos, indicating a loss of Nrg1 expression (Fig 2C). Our gene editing strategy is expected to eliminate both *nrg1-I* and *nrg1-II*, while sparing the *Nrg1-III* isoform. It is important to note that functional Nrg1 protein could not be assessed via Western blot due to a lack of available Nrg1 antibody. Previous report on the *nrg1*^*z26*^ allele, which codes for a loss of function mutation in the CRD domain of *nrg1-III*, demonstrated that loss of *nrg1-III* leads to supernumerary neuromasts in the developing lateral line as well as later adult lethality [55]. To determine if *nrg1-III* signaling is intact in our mutants, we used a voltage sensitive vital dye (Mitotracker) to label neuromasts in embryos at 5 dpf [31,56] (Fig 2D). Though *nrg1*^*z26*^ mutants had supernumerary neuromasts, extra neuromasts were not observed in offspring of inbred *nrg1*^*WT/nc28*^, indicating that *nrg1*^*nc28*^ does not disrupt the function of *nrg1-III* (Fig 2F).

### Trabeculae form in *nrg1*^*nc28*^ mutants in an ErbB2-dependent manner

**Fig 3:**
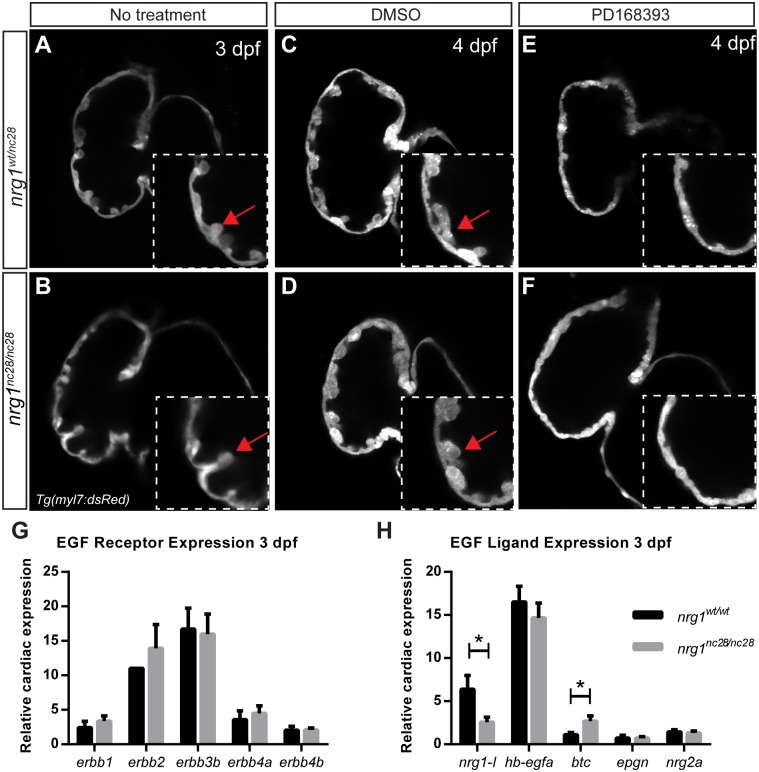
*nrg1* mutants require ErbB2 tyrosine kinase activity to form trabeculae. (A-F) Representative confocal optical mid-chamber slice of the ventricle at 3–4 dpf in larvae carrying *Tg(myl7*:*dsRed)* cardiomyocyte reporters. Boxes include high-resolution image of the outer curvature. Larvae were examined at (A-B) 3 dpf or (C-F) 4 dpf after treatment with (C-D) 1% DMSO or (E-F) 3.75 μM PD168393 from 2–4 dpf. Larvae were genotyped after imaging. Red arrows point to representative trabeculae. N ≥ 4 larvae for each condition and genotype. Relative expression levels of (G) EGF family receptor genes or (H) EGF family receptor ligand genes from isolated hearts of *nrg1*^*WT/WT*^ and *nrg1*^*nc28/nc28*^ larvae at 3 dpf. N = 3–5 biological replicates with 30–60 hearts pooled per condition normalized to *efl1a*. N = 1 biological replicates with 30–60 hearts pooled for *erbb2* normalized to *efl1a*. Student’s T-test mutant compared to wild type. Error bars are SEM. N≥3 biological replicates. *p≤0.05–0.01.

To address the hypothesis that *nrg1-I* is required for stimulating cardiac trabeculation, we crossed the *nrg1*^*nc28*^ allele onto a transgenic background expressing dsRed in cardiomyocytes. We used confocal microscopy to examine cardiac trabeculation in the *nrg1*^*WT/nc28*^ and *nrg1*^*nc28/nc28*^ fish from 2–5 dpf. Trabeculation has been previously shown to start around 58 hpf [31,57]. At 2 dpf, trabeculae were undetectable in both genotypes, suggesting that *nrg1-I* does not negatively regulate initiation of trabeculae. However, at 3 dpf trabeculae were detectable and indistinguishable between genotypes (Fig 3A and 3B). To verify that trabeculation in *nrg1*^*nc28*^ mutants was not due to escape from requirement of ErbB2 signaling, we incubated embryos from 2 to 4 dpf with the ErbB2-tyrosine kinase specific inhibitor PD168393 or vehicle and observed inhibition of trabeculation in both *nrg1*^*WT/nc28*^ and *nrg1*^*nc28/nc28*^ fish (Fig 3C–3F). Together, our data suggest that *nrg1-I* is dispensable for the initiation of cardiac trabeculation and trabeculation still occurs through the ErbB2 pathway in our *nrg1*^*nc28*^ mutants.

Since other EGF-like ligands are predicted to have affinity for ErbB2/ErbB4 heterodimers, we hypothesized that other EGF-like ligands can compensate for a loss of *nrg1-I*. We screened *nrg1*^*WT/WT*^ and *nrg1*^*nc28/nc28*^ hearts at 3 dpf for expression of known EGF-like ligands and receptors (S1 Table). Transcripts for ErbB receptors *egfr1* (*erbb1*), *erbb2*, *erbb3b*, and *erbb4* were detectable and expressed at comparable levels between all genotypes (Fig 3G). Five EGF-like ligands, *nrg1-I*, *heparin-binding egf-like receptor a* (*hb-egfa*), *neuregulin 2a* (*nrg2a*), *betacellulin* (*btc*) and *epigen* (*epgn*) were also detected (Fig 3H). As expected for nonsense-mediated decay, *nrg1-I* transcripts were reduced in mutant hearts (Fig 3H). Interestingly, *btc* transcript levels were slightly elevated in mutant hearts at 3 dpf, suggesting a possible compensatory role for *btc* (Fig 3H). However, additional studies are necessary to distinguish between an absolute requirement and a compensatory role for each of these ErbB2/ErbB4-activating ligand(s) in trabeculation.

### The adult *nrg1*^*nc28*^ mutant does not show overt morphological defect

**Fig 4:**
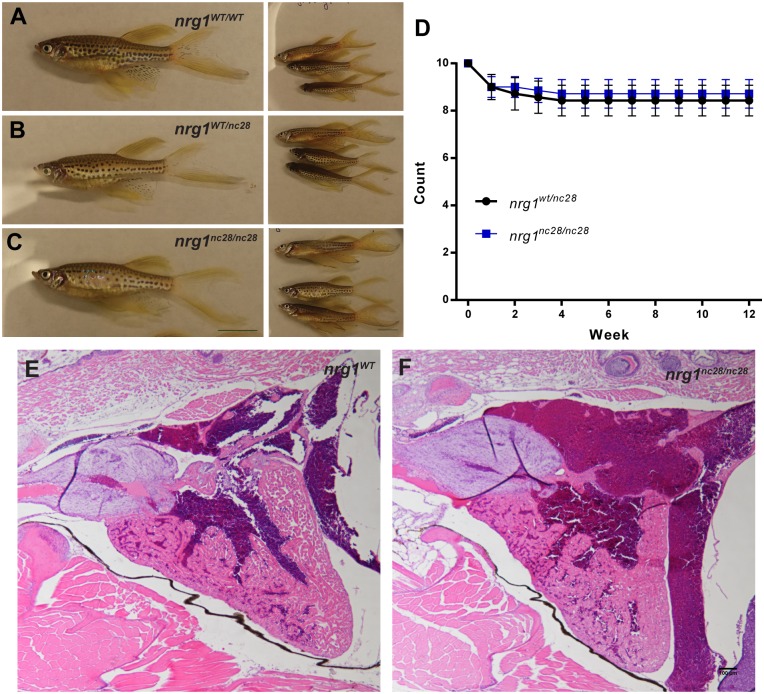
*nrg1*^*nc28*^ survive to adulthood without overt cardiac abnormalities. (A-C) Representative gross morphology of age-matched (A) *nrg1*^*WT/WT*^, (B) *nrg1*^*WT/nc28*^ or (C) *nrg1*^*nc28/nc28*^ clutch mates, Standard Length (SL) 25–30. Scale bar is 10mm. (D) Weekly survival of fish from sibling *nrg1*^*WT/nc28*^ and *nrg1*^*nc28/nc28*^. (E-F) Representative cross section of the heart in H&E stained section of formaldehyde-fixed, paraffin embedded (E) *nrg1*^*WT/WT*^ and (F) *nrg1*^*nc28/nc28*^adult fish. BA = Bulbous Arteriosus, V = Ventricle, A = Atrium. N = 3 fish per genotype. Scale bar 100 μm.

Though our findings indicate that Nrg1 is dispensable for cardiac morphogenesis through larval stages, Nrg1 may be involved in other developmental processes. To address this possibility, we interbred heterozygous fish and followed sibling offspring to adulthood. Homozygous mutant *nrg1*^*nc28*^ fish were indistinguishable from wild type or heterozygous clutch mates at the gross morphological level (Fig 4A–4C). Similarly, survival from larval to early adulthood was comparable between genotypes (Fig 4D). To explore the possibility that *nrg1-I* and *nrg1-II* isoforms may modulate cardiac morphology in a non-lethal manner, we examined the H&E stained heart sections of *nrg1*^*wt/wt*^ and *nrg1*^*nc28/nc28*^ adult clutch mates and found no obvious differences in gross morphology (Fig 4E and 4F). Together, our findings indicate that *nrg1-I* and *nrg1-II* is dispensable for development and homeostatic function in zebrafish within the limit of our phenotypic analysis.

## Discussion

This study highlights an example of cross-species differences in EGF family member requirements for the process of cardiac trabeculation. Previous mouse studies have shown that *nrg1-I* has a critical role in trabecular development [7]. More specifically, knockout studies have demonstrated that loss of *nrg1-I* and *nrg1-II* isoforms causes embryonic lethality likely due to a complete loss of cardiac trabeculation and subsequent defect in cardiac contractility and function [58]. In contrast, in zebrafish, our study suggests that loss of *nrg1-I* and *nrg1-II* function does not have any survival or phenotypic consequences under homeostatic conditions. The zebrafish *nrg1-I* and *nrg1-II* mutant still develops cardiac trabeculae and survives to adulthood without overt cardiac abnormalities.

Unfortunately, we were unable to assess functional Nrg1 protein levels via Western blot in this study due to unavailability of Nrg1antibody. Despite this obstacle, our assays of pharmacological inhibition of ErbB2 activity and gene expression data of other EGF-like ligands suggests cardiac trabeculation occurs in zebrafish *nrg1-I/II* mutant in an ErbB2-dependent manner. Pharmacological inhibition of ErbB2 activity in both WT and the *nrg1-I/II* mutant completely abolished trabecular formation. These results suggest that other *nrg1-I*-like factors could function as the major ligand for ErbB4 receptor, act redundantly with *nrg1-I* to regulate cardiac trabeculation, or may act in a compensatory manner when *nrg1-I* is absent. Except for *nrg1-I*, we found that another four EGF-like ligands encoded by *hb-egfa*, *btc*, *epgn* and *nrg2a* were also expressed in the zebrafish heart during trabeculation stage. The question arises as to which EGF-like ligand(s) could replace the function of or compensate for the loss of *nrg1-I* to regulate cardiac trabeculation in zebrafish. We suggest that the zebrafish genomic structure supports divergent evolution of EGF-like ligands in regulating cardiac trabeculation. Therefore, ligands with paralogs in the zebrafish genome are the most likely candidates for this function.

The zebrafish genome contains gene duplications due to the teleost-specific whole genome duplication event during teleost speciation. This type of gene duplication allows one of the paralogues to evolve new functions while the other retains the gene’s original function [44,59]. The zebrafish genome contains paralogues of *hbegfa* and *nrg2a*, *hbegfb* and *nrg2b*, it is thus possible that either *hbegfa* or *nrg2a* acquired a role in regulating cardiac trabeculation that could compensate or supersede any role that *nrg1* may play in trabeculation. Alternatively, since *btc* transcript levels were elevated in *nrg1-I*/*II* mutant, the upregulation of *btc* expression may compensate for the loss of *nrg1-I* to regulate cardiac trabeculation in zebrafish. Nevertheless, mutants that ablate the function of these Nrg1 like factor(s) will need to be generated to determine whether and which ligand(s) have the primary role of regulating trabeculation and what role Nrg1 plays in the developing zebrafish heart.

Our study highlights the differences and complexity of zebrafish *nrg1-I* function relative to other model species, specifically in the process of cardiac trabeculation. In rodent models, loss of *nrg1-I* is developmentally lethal. Conversely, our work demonstrates that zebrafish do not share the same conserved role for *nrg1-I*. Additionally, we have highlighted the need to further define the roles of all Nrg1 isoforms in cardiac development and trabeculation within the zebrafish model system. While the function of *nrg1-I* and *nrg1-II* in cardiac trabeculation might be replaced by EGF-like ligands in the zebrafish genome, the third *nrg1* isoform *nrg1-III* appears to play an evolutionary conserved role in Schwann cell development [7,60–62]. In zebrafish, the *nrg1*^*z26*^ mutant specifically disrupts *nrg1-III* function, leading to severe defects in Schwann cell migration and proliferation [55]. The differences in phenotypic consequences upon ablating the function of different Nrg1 isoforms suggest that the function of the different Nrg1 isoforms could evolve independently in different tissue and cell types.

## Supporting Information

S1 Fig Predicted translations of *nrg1-I* mutant alleles. (A) *nrg1-I*^*WT*^ allele is translated into 599 amino acid (aa), (B) *nrg1-I*^*nc28*^ into 55 aa, (C) *nrg1-I*^*nc29*^ into 99 aa. (B-C) Amino acids that differ from wild type are in red. Asterisk indicates stop codon.
(TIF)

S1 Table List of Primers used in this study. (DOCX)
