# Supplementary figures and images for: IgG-Containing Isoforms of Neuregulin-1 Are Dispensable for Cardiac Trabeculation in Zebrafish
Source: PLoS One. 2016 Nov 15;11(11):e0166734. doi: 10.1371/journal.pone.0166734 (PMC5112773; doi:10.1371/journal.pone.0166734)

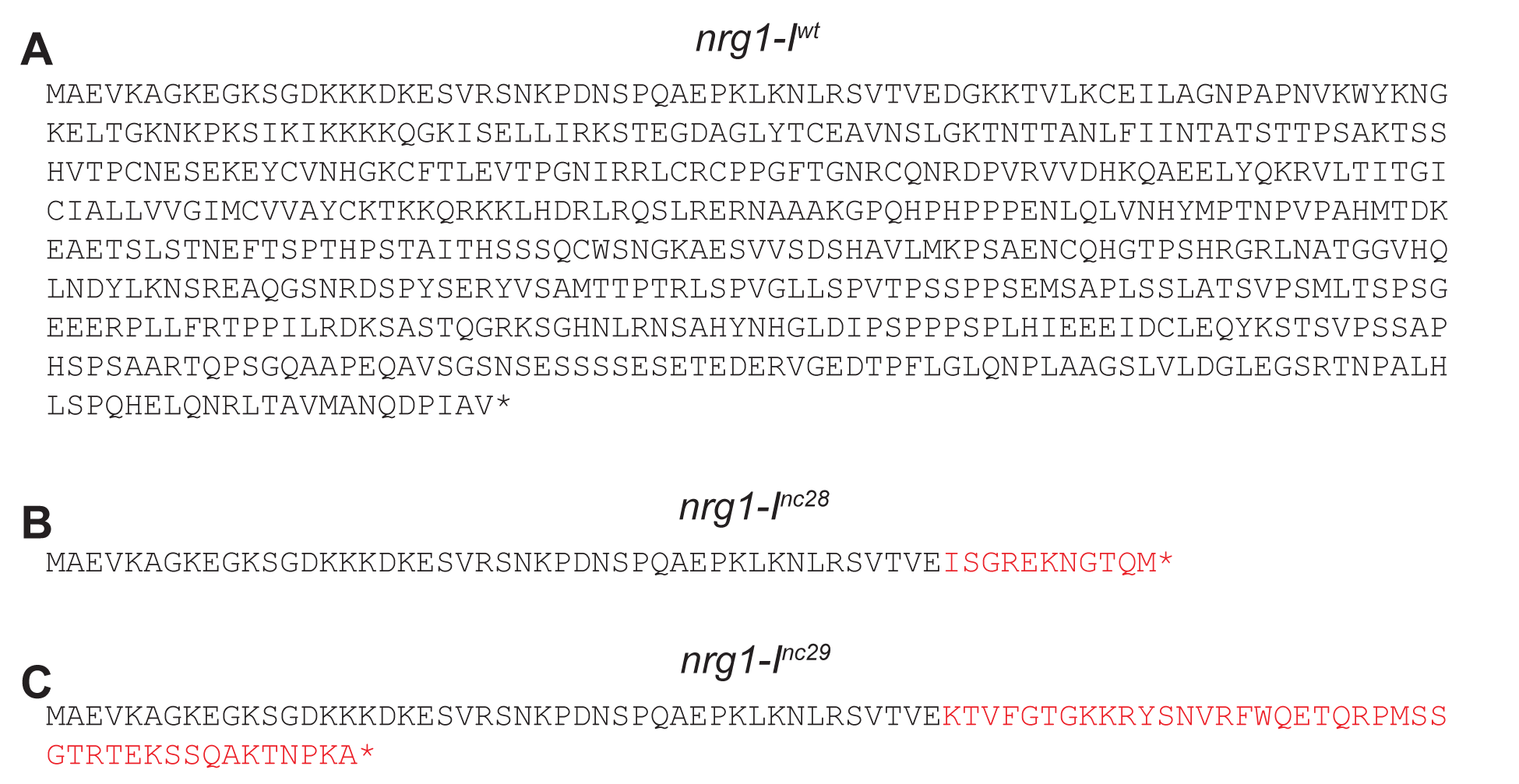

Supplement: S1 Fig — (A) nrg1-IWT allele is translated into 599 amino acid (aa), (B) nrg1-Inc28 into 55 aa, (C) nrg1-Inc29 into 99 aa. (B-C) Amino acids that differ from wild type are in red. Asterisk indicates stop codon. (TIF) [file pone.0166734.s001.tif]
