# Supplementary material for: IgG-Containing Isoforms of Neuregulin-1 Are Dispensable for Cardiac Trabeculation in Zebrafish
Source: PLoS One. 2016 Nov 15;11(11):e0166734. doi: 10.1371/journal.pone.0166734 (PMC5112773; doi:10.1371/journal.pone.0166734)
Supplement: S1 Table — (DOCX) [file pone.0166734.s002.docx]

**Table S1: Primer sequences**

| Target | Use | Forward Oligo | Reverse Oligo |
| --- | --- | --- | --- |
| *nrg1-exon3* | *HRMA* | TTTTCCAGCGGAACCGAAGT | GTACCACTTGACATTGGGCG |
| *nrg1-E10-E14* | PCR | CCCTCGGCCAAGACATCC | TTGCGGTGAGAGGTGCAG |
| *ef1a* | qRT-PCR | AGGACATCCGTCGTGGTAAT | AGAGATCTGACCAGGGTGGTT |
| *areg* | qRT-PCR | GAACACATCATCGCTTCCAGG | AAACCCGTCCATCAGTGACTT |
| *btc* | qRT-PCR | GGACACTTCTCTGCCTGTCC | TTTTCGCATCTGCATGACGG |
| *egf* | qRT-PCR | GTTTCAGCTGTCAAGCAGAGTT | GCACGCCATTTTTGTGTTGC |
| *epgn* | qRT-PCR | GTCAGAAGTCACCACCACCC | CGTCTCACTTGTGGAGTCGT |
| *hb-egfa* | qRT-PCR | TCCTGGCACTCAGGTTTCAC | TGGTTTAGTGGTGTGGAGCG |
| *hb-egfb* | qRT-PCR | TGCTTTTCTGACAGGTACCAC | TTTTGGCTTTCTTTATCCTTCGTT |
| *nrg1-I* | qRT-PCR | GTGAAAGCAGGCAAAGAAGGG | TGTCACGCTCCGAAGGTTTT |
| *nrg1-III* | qRT-PCR | ACCCACAAATGACACGTCCG | ACTGTCACGCTCCGAAGGTT |
| *nrg1-III* | qRT-PCR | CAGCCCCAAGAGCACCTTT | GTGACTGGATGTCTTGGCCG |
| *nrg2a* | qRT-PCR | GCCGGCAACTGAGAGAGTAAT | CGCTGACACATACCTGTGGG |
| *nrg2b* | qRT-PCR | ATCCTGTCTCAGCATGAGGG | TTTGGAGCTGATGCCCTCTTTT |
| *nrg3* | qRT-PCR | AAAACACGGTAATGCCGAAGC | CAAGACCTCAAGCAAAACAGACA |
| *tgfa* | qRT-PCR | TGTATGCCATCCTGGCTTTGT | TCCACCAACAAAACACCAGC |
| *erbb1* | qRT-PCR | ATGGGCCTTTCTGAACCCAG | CTCTACTGGCATCACGGGAC |
| *erbb2* | qRT-PCR | GACTTCACTGCTCCACCCAA | CCCAACAACCTGAATCCCCA |
| *erbb3a* | qRT-PCR | TGAACATTCAGTCTTGGCCCG | TCATCAACATAGAGAATGGCGTGT |
| *erbb3b* | qRT-PCR | ACCTTGTGGTGAGGCCTGCTC | CGCAAACCCAACCTGCAACC |
| *erbb4a* | qRT-PCR | ACATCCTGGAGAAAGGCGAACGT | CTCGTGCCATACGGCTGAACTCT |
| *erbb4b* | qRT-PCR | TGGGTTCCTGAGGGTGAGACTG | ATGCTGGCCATGATCAGAGCCT |
